# Transfer of exosomal microRNA-203-3p from dendritic cells to bone marrow-derived macrophages reduces development of atherosclerosis by downregulating Ctss in mice

**DOI:** 10.18632/aging.103842

**Published:** 2021-06-02

**Authors:** Beiyou Lin, Wenchao Xie, Chunmei Zeng, Xiaodan Wu, Ang Chen, Hao Li, Rina Jiang, Ping Li

**Affiliations:** 1 Department of Cardiology, Yulin First People’s Hospital and The Sixth Affiliated Hospital of Guangxi Medical University, Yulin 537000, P.R. China; 2 Department of Cardiology, The First Affiliated Hospital of Guangxi Medical University and Guangxi Key Laboratory Base of Precision Medicine in Cardio-Cerebrovascular Diseases Control and Prevention and Guangxi Clinical Research Center for Cardio-Cerebrovascular Diseases, Nanning 530021, P.R. China

**Keywords:** dendritic cells, exosomes, microRNA-203-3p, atherosclerosis, cathepsin S

## Abstract

Dendritic cell-derived exosomes have been proven to be efficient adjuvant options for anti-tumor vaccines in cancer immunotherapy. However, their potency in atherosclerosis remains unclear. Here we summarize the association of microRNA-203-3p (miR-203-3p) with dendritic cell-derived exosomes and atherosclerosis. Firstly, dendritic cell-derived exosomes and bone marrow-derived macrophages were isolated, after which expression of miR-203-3p and cathepsin S was determined. After the establishment of atherosclerosis mouse models, gain- and loss-of-function experiments were conducted for the analysis of effects of miR-203-3p and cathepsin S on foam-cell formation, lipid accumulation, collagen deposition and serum total cholesterol. The results found high expression of cathepsin S in atherosclerosis mice and downregulation of miR-203-3p in the serum of atherosclerosis patients and ox-LDL-simulated bone marrow-derived macrophages. Cathepsin S was the target gene of miR-203-3p. miR-203-3p transporting from exosomes to bone marrow-derived macrophages resulted in inhibition of cathepsin S expression and atherosclerosis-related phenotypes in bone marrow-derived macrophages, thus alleviating atherosclerosis in mice, and this process was found to involve the p38/MAPK signaling pathway. These findings provided evidence that the transfer of miR-203-3p by dendritic cell-derived exosomes targeted cathepsin S in bone marrow-derived macrophages to attenuate atherosclerosis progression in mice, serving as a promising clinical target for atherosclerosis.

## INTRODUCTION

Atherosclerosis (AS) is a condition characterized by the narrowing and hardening of blood vessels that occurs secondary to plaque buildup around arterial walls. AS typically takes decades to reach advanced stages, at which point it can result in artery disease, chest pain, stroke or even cardiac arrests [1]. The underlying pathophysiology of AS includes the gathering of apolipoprotein B-lipoproteins in the matrix underneath the endothelial cell of blood vessels, which leads to the accumulation of monocytes which then differentiate into macrophages and dendritic cells (DCs). This accumulation of apoptotic foam macrophages and elevated regional inflammation response weaken vessel walls and facilitate thrombus formation, thus leading to atherosclerosis (AS) [2]. Therefore, monocyte-derived cells have been emphasized in increasing studies as they are capable of effectively treating AS. There’s been an increase in AS therapies that are able to regulate macrophage content by lowering macrophage recruitment to atherosclerotic plaques, and enhancing macrophage apoptosis as well as emigration, the goal of which is to reduce formation of plaques [3]. DCs are present in healthy arteries and accumulate at the sight of the atherosclerotic lesions during AS formation, suggesting that they play both pathogenic and protective roles in AS [4]. In addition, DC-derived exosomes (DCexs) have received a great deal of recognition as anti-cancer agents since they possess functional major histocompatibility complex-peptide complexes and other immune-stimulating components, which enhance immune cell-dependent tumor rejection [5]. For example, exosome-shuttle microRNAs (miRNAs) have been previously demonstrated to mediate DC functions [6]. However, the underlying mechanism by which miRNAs delivered by DCexs are involved in the progression of atherosclerosis remains unclear.

miRNAs, about 23 nt in length, play imperative gene-regulatory roles in animals and plants by pairing with the mRNAs of protein-coding genes or lncRNA genes to guide their post-transcriptional regulation [7]. Additionally, miRNAs are known to regulate vascular smooth muscle cells and endothelial cells (ECs) as well as macrophage functions, thus modulating the development of AS. A diverse set of stimuli can regulate miRNA expression, which takes part in each stage of the progression of AS; therefore, numerous circulating miRNAs have been identified as biomarkers for AS staging [8]. For instance, reduced miR-145 promoted lesion formation by controlling the differentiation of smooth muscle cells, whereas the EC-specific miR-126 induced endothelial repair through its transmission from apoptotic ECs in micro-vesicles [9]. In particular, co-inertia analysis, a new computation method revealed that miR-203 was associated with early AS [10]. Moreover, another study also found that upregulated levels of miR-203 could reduce atherosclerotic lesion area in Ang II ApoE(-/-) mice [11]. Moreover, bioinformatics analysis conducted in the present study reported that miR-203-3p targeted cathepsin S (Ctss). Human atherosclerotic and aneurysmal tissues have a high expression of Ctss, a lysosomal protease that may be responsible for inflammatory diseases [12]. Therefore, selective suppression of Ctss with aims of reducing the inflammatory response has also been highlighted as a practicable therapeutic option for AS, providing further verification that AS is an inflammatory disease involved with multiple immune cells [13]. Moreover, down-regulation of Ctss induced by methyl protodioscin could induce human oral cell autophagy and apoptosis through the mediation of the p38/mitogen-activated protein kinase (MAPK) signaling pathway [14]. MAPK is an important factor of cellular signal transduction, particularly in mediating inflammatory genes in the progression of atherosclerosis [15]. The p38/AMPK signaling pathway has also been implicated in the activation of inflammasome in rats with AS, as evidenced by significantly elevated protein expression of p-p38 MAPK in the AS rats [16]. Based on the aforementioned findings and studies, we hypothesized that miR-203-3p might be delivered by exosomes derived from DCs, after which it regulates the formation of atherosclerotic lesions via Ctss. Therefore, bone marrow-derived macrophages (BMDMs) and DC-derived exosomes were extracted and AS animal models were established to confirm this hypothesis in the present study.

## RESULTS

### Ctss is highly expressed and miR-203-3p is poorly expressed in AS

**Figure 1:**
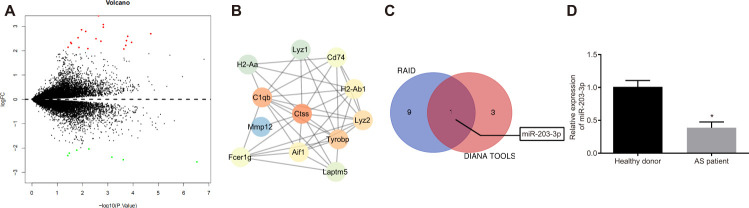
**The elevation of Ctss and decline of miR-203-3p are identified in AS.** (**A**) The volcanic map of DEGs between from GSE56143 with -log10 logarithm of *p* value as the abscissa and logFC multiple as the ordinate, and |logFC| > 2 as threshold, where 19 genes are up-regulated and indicated as red, and 7 genes are down-regulated and indicated as green. (**B**) Protein interaction map of DEGs in which the brighter color indicates the higher core level. (**C**) Online prediction of miRNAs that target Ctss. (**D**) miR-203-3p expression in DCexs determined by RT-qPCR. ^*^ *p* < 0.05 *vs.* the healthy volunteers. Statistical data were measurement data, and described as mean ± standard deviation. The paired *t* test was used for comparison between two groups. n = 76 for AS patients, while n = 49 for healthy volunteers.

The differentially expressed genes in AS (Figure 1A, Supplementary Table 1) were screened from the GSE56143 dataset using the R language, and the protein interaction was analyzed by the String database (https://string-db.org/). Ctss gene was identified as a critical gene in AS mice, due to its significantly high expression (Figure 1B). In order to explore the molecular mechanism of Ctss implicating microRNA in AS, we employed RAID (http://www.rna-society.org/raid/index.html) and DIANA TOOLS (http://diana.imis.athena-innovation.gr/DianaTools/index.php?r=microT_CDS/index) for the prediction of miRNAs that bind to Ctss and discovered that there were binding sites between miR-203-3p and Ctss. RAID predicted 10 Ctss-bound miRNAs, while the DIANA TOOLS predicted 4 Ctss-bound miRNAs, and 1 miRNA (miR-203-3p) was identified as the intersection between the two softwares (Figure 1C). In addition, DCs have been implicated in various pathogenic and protective mechanisms during AS [4], while miR-203-3p expression was found in the DCexs [6] and exerted an inhibitory role on vascular calcification [17]. In accordance with the aforementioned, the expression of miR-203-3p in the serum of AS patients was lower compared to that in healthy volunteers (Figure 1D; *p* < 0.05). These results suggest that the expression of miR-203-3p was significantly reduced, while that of Ctss was evidently elevated in AS.

### *In vitro* cell model is successfully established and the DCexs are successfully extracted

**Figure 2:**
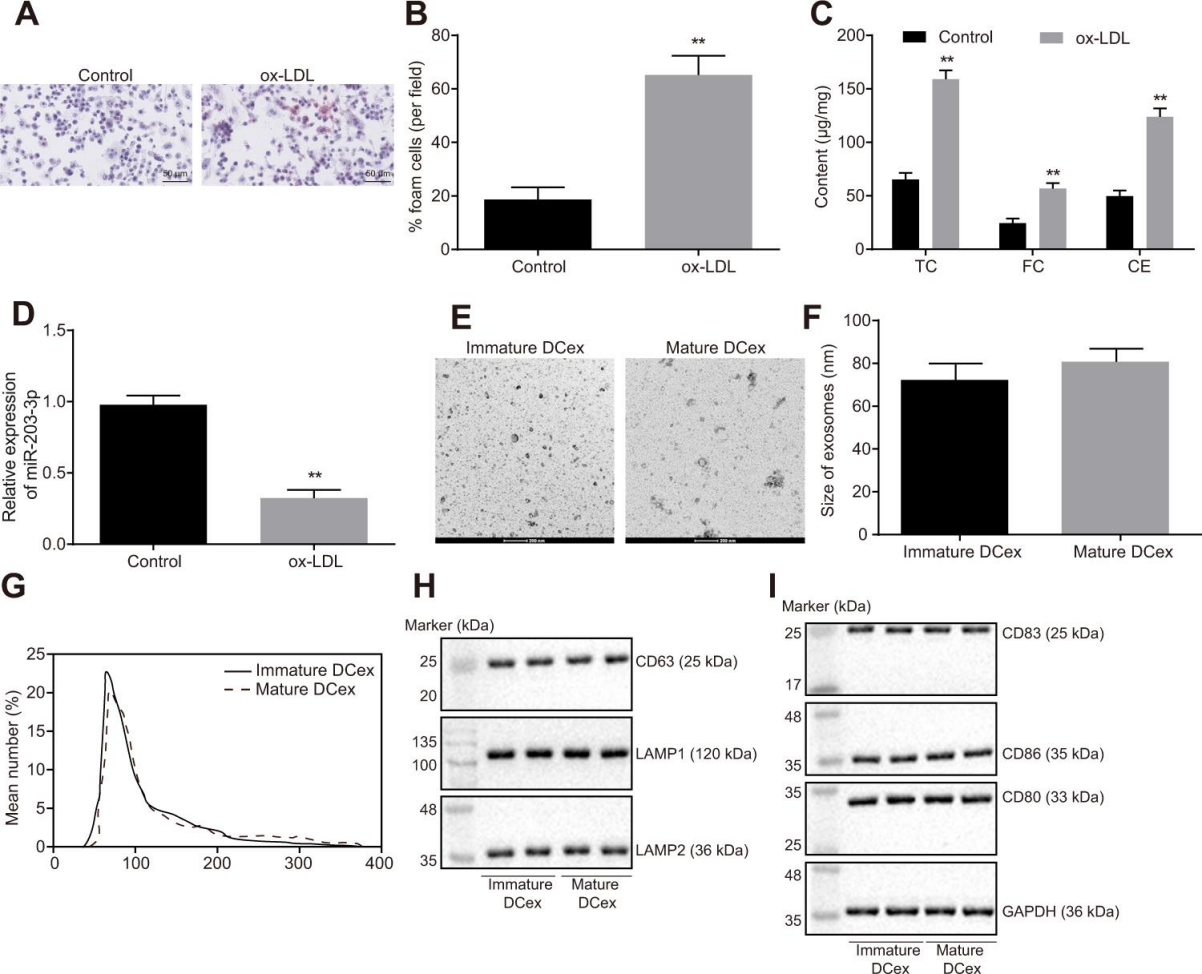
**ox-LDL induction of BMDM and identification of DCex.** (**A**, **B**), The proportion of foam cells in BMDMs by oil Red O staining (magnification: 200 ×). (**C**) Serum TC, FC and CE levels determined by ELISA. (**D**) miR-203-3p expression measured by RT-qPCR. (**E**, **F**) Identification of the structure and size of exosomes by transmission electron microscopy (scale bar = 200 nm). (**G**) Diameter and number of exosomes assessed by Nanosight particle tracking analysis. (**H**–**I**) The expression of exosomes markers (CD63, LAMP1 and LAMP2) and DC markers (CD83, CD86 and CD80) evaluated by Western blot analysis (50 μg protein was loaded). Statistical data were measurement data, and described as mean ± standard deviation. The paired *t* test was used for comparison between two groups in panel (**A**–**F**). The experiment was repeated 3 times independently to obtain the mean value.

Foam cells are pro-necrotic M2 macrophages and the early feature of formation of atherosclerotic lesion [18]. Firstly, BMDMs were induced with ox-LDL, and the proportion of BMDM-derived foam cells were examined using oil red O staining. The findings showed that ox-LDL promoted the development of BMDMs into foam cells (Figure 2A, 2B; *p* < 0.05). The levels of TC, FC and CE were all found to be increased significantly (Figure 2C; *p* < 0.01). Additionally, the results of reverse transcription quantitative polymerase chain reaction (RT-qPCR) showed significantly reduced miR-203-3p expression after ox-LDL intervention (Figure 2D; *p* < 0.05).

There was no significant difference in size and number of immature and mature DCexs, whereas the quantification results of exosome markers CD63, LAMP1 and LAMP2 indicated that DC-derived particles collected in our experiments were identified as exosomes (Figure 2E–2H). Western blot analysis results showed that DCexs expressed specific antigens such as CD83, CD86 and CD80 (Figure 2I).

### DCexs inhibit BMDM phenotypes of AS

**Figure 3:**
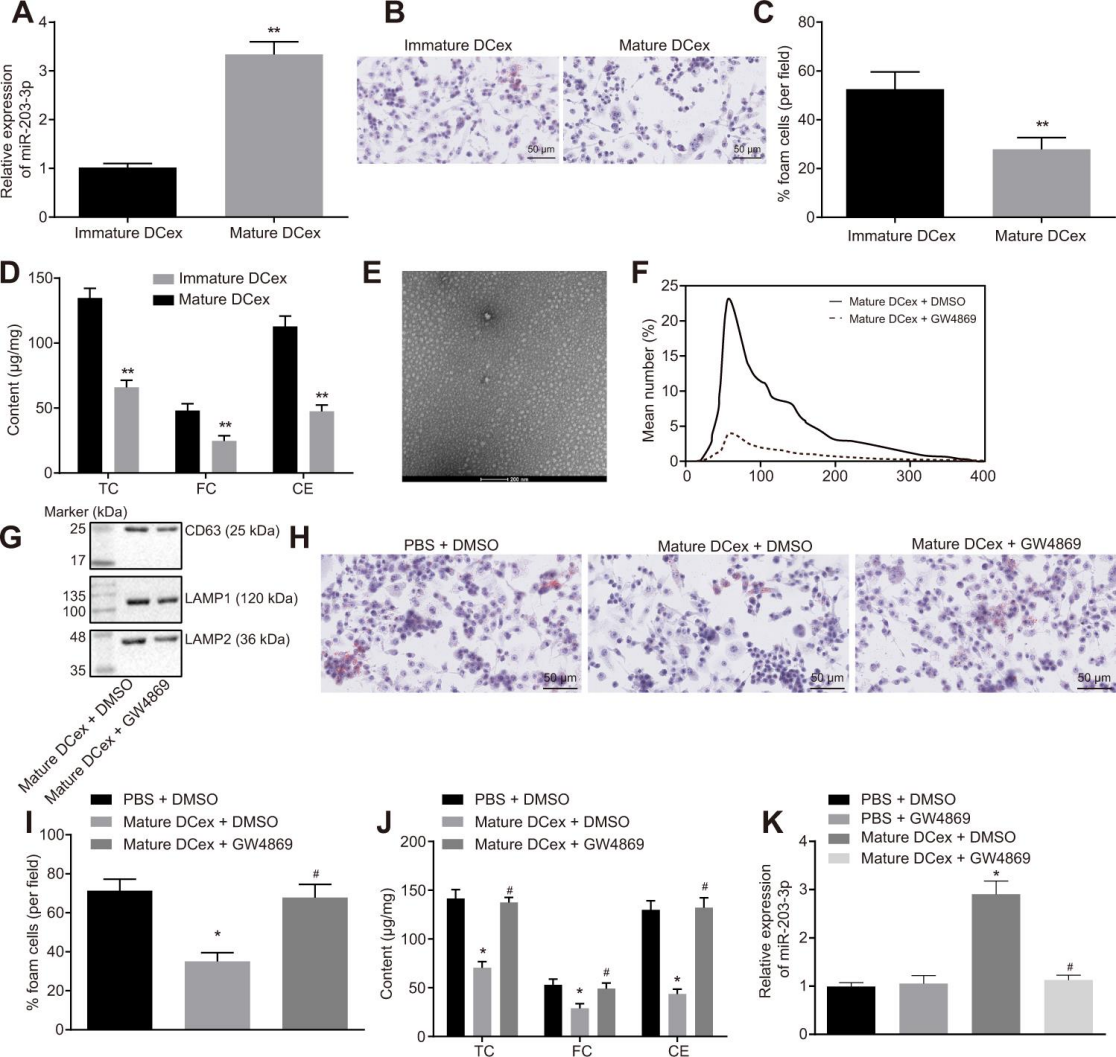
**BMDM phenotypes of AS are repressed by DCex.** BMDMs were initially treated with ox-LDL or without treatment. (**A**) miR-203-3p expression in immature and mature exosomes from DCs by RT-qPCR. (**B**, **C**) The proportion of foam cells in immature and mature exosomes from DCs by oil red O staining (200 ×). (**D**) TC, FC and CE levels in immature and mature exosomes from DCs determined by ELISA. (**E**) Morphology of exosomes after GW4869 treatment (scale Bar = 200 nm) detected by transmission electron microscopy. (**F**) Nanosight particle tracking analysis of the size and quantity of exosomes. (**G**) Western blot analysis of the expression of exosome marker proteins. (**H**–**I**) The proportion of foam cells in BMDMs after 24-h stimulation with ox-LDL measured by oil red O staining (200 ×). (**J**) Serum TC, FC and CE levels in BMDMs after 24-h stimulation with ox-LDL determined by ELISA. (**K**) miR-203-3p expression in BMDMs determined by RT-qPCR. ^*^ *p* < 0.05 and ^**^ *p* < 0.01 *vs.* the BMDMs without treatment; ^*^ *p* < 0.05 *vs.* PBS + DMSO; ^#^ *p* < 0.05 *vs.* DCex + DMSO. Statistical data were measurement data, and described as mean ± standard deviation. The paired *t* test was used for comparisons between two groups in panel (**A**–**D**). The one-way analysis of variance was adopted for comparisons among multiple groups in panel (**I**–**K**) followed by Tukey’s post hoc test. The experiment was repeated 3 times independently.

To further investigate the effect of DCexs on BMDMs, miR-203-3p expression was examined in DCexs at different mature levels using RT-qPCR. The results showed that miR-203-3p expression was up-regulated in mature DCexs (Figure 3A; *p* < 0.05). Co-culture of DCexs with ox-LDL-treated BMDMs revealed that the AS phenotype of BMDMs in mature DCexs was inhibited, as evidenced by reduced FC proportion, TC, FC and CE levels when compared to immature DCexs (Figure 3B–3D; *p* < 0.05). Subsequent studies were conducted on mature DCs.

Additionally, mature dendritic cells received treatment with GW4869 (an exosome secretion inhibitor), and then AS phenotype of BMDMs stimulated by ox-LDL was detected. It was found that the number of exosomes was significantly decreased under transmission electron microscopy (Figure 3E). NTA analysis (Figure 3F) illustrated that the size and quantity of exosomes did not change significantly, while the number of exosomes presented with a sharp decline. Meanwhile, the exosome marker proteins, CD63, LAMP1 and LAMP2, were all found to be markedly decreased (Figure 3G). In addition, we found that GW4869 increased BMDM-derived foam cells and levels of TC, FC and CE, but reduced the relative expression of miR-203-3p (Figure 3H–3J; *p* < 0.05). Simultaneously, DCexs were noted to up-regulate miR-203-3p expression in the BMDMs (*p* < 0.05). In addition, compared with PBS + DMSO treatment, miR-203-3p expression showed no significant difference after treatment with PBS + GW4869. Compared with treatment with DCex + DMSO mi-203-3p expression was obviously diminished after DCs were treated with GW4869 (*p* < 0.05), while there was no change of miR-203-3p expression after treatment with PBS+DMSO (Figure 3K; *p* > 0.05). Thus, DCexs might play an essential role in the suppression of the phenotypes of BMDMs in AS.

### miR-203-3p transferred from DCexs inhibits BMDM phenotypes related to AS

**Figure 4:**
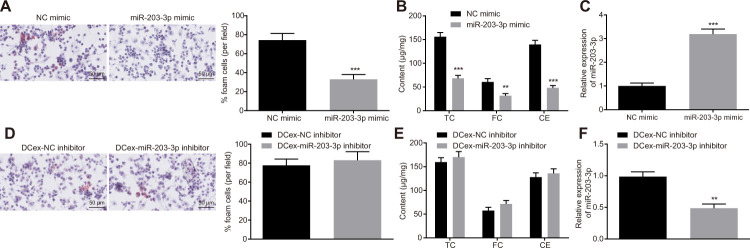
**Overexpression of miR-203-3p in DCexs impairs AS-related phenotypes in BMDMs.** BMDMs were treated with miR-203-3p mimic with NC mimic as the control, or treated with DCex-miR-203-3p inhibitor with DCex-NC inhibitor as control. (**A** and **D**) The proportion of foam cells in BMDMs by oil red O staining (200 ×). (**B** and **E**) Serum TC, FC and CE levels determined by ELISA. (**C** and **F**) miR-203-3p expression measured by RT-qPCR; ^*^ *p* < 0.05, ^**^ *p* < 0.01, ^***^ *p* < 0.001 *vs.* the BMDMs treated with NC mimic or DCexs treated with NC inhibitor. Statistical data were measurement data, and described as mean ± standard deviation. The paired *t* test was used for comparisons between two groups. The experiment was repeated 3 times independently.

To explore the functional role of miR-203-3p in AS, both gain and loss-of-function experiments were performed in BMDMs treated with ox-LDL. After miR-203-3p mimic was introduced, FC formation as well as TC, FC and CE levels was found to be significantly reduced in BMDMs (Figure 4A, 4B, 4D, 4E; *p* < 0.05), while the expression of miR-203-3p was significantly restored (Figure 4C, 4F; *p* < 0.05). Following the inhibition of miR-203-3p expression in DCs, exosomes were extracted and co-cultured with BMDMs stimulated by ox-LDL. Consequently, DCs lost the ability to inhibit the atherosclerotic phenotype of BMDMs after inhibiting miR-203-3p. These findings suggest that miR-203-3p could inhibit AS-related phenotypes in BMDMs.

### Ctss is the target of miR-203-3p in AS

**Figure 5:**
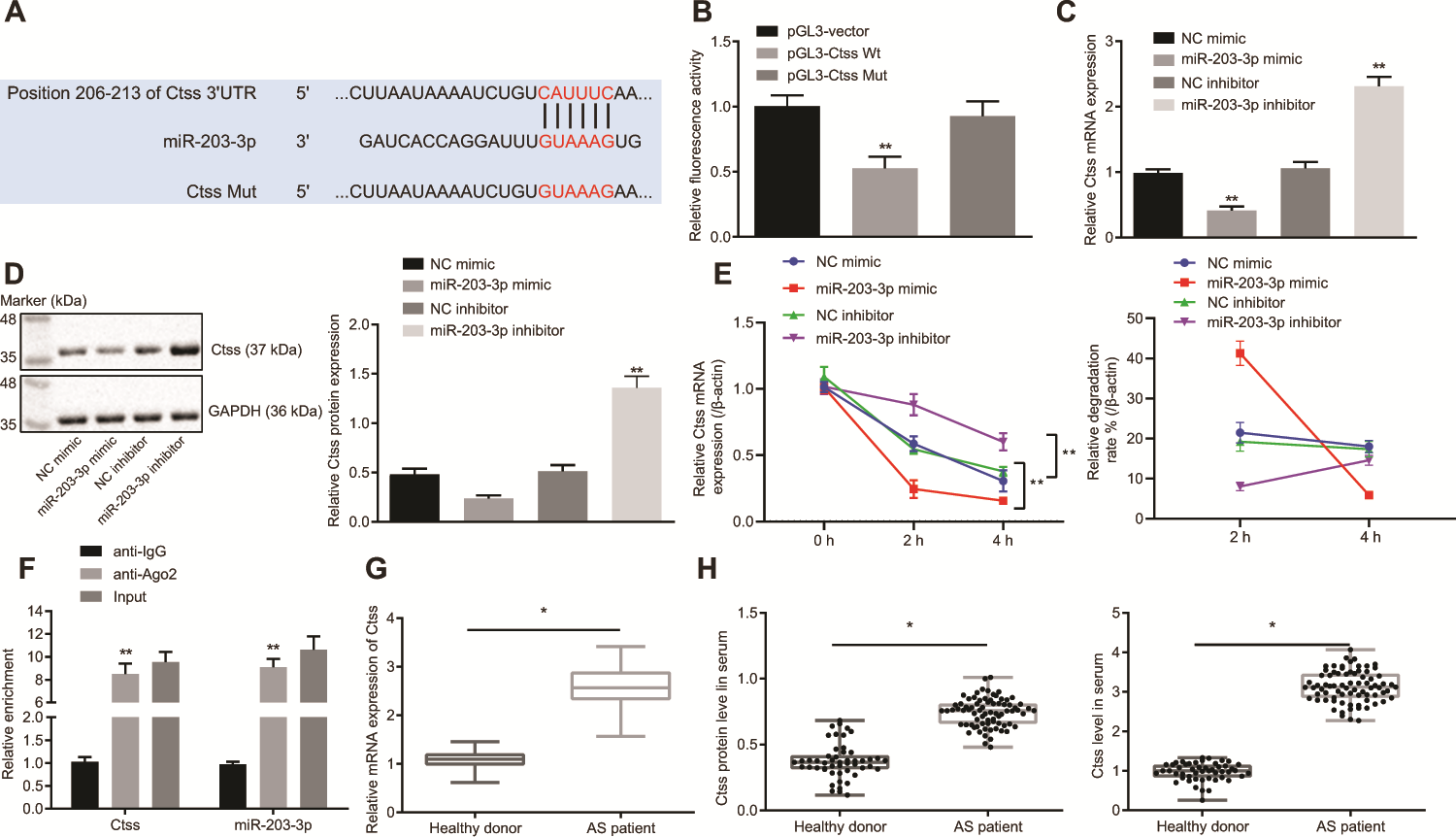
**Validation of the relationship between miR-203-3p and Ctss.** (**A**) TargetScan prediction of miR-203-3p-Ctss binding sites. (**B**) The binding of miR-203-3p to Ctss confirmed by dual-luciferase reporter gene assay. (**C**) mRNA expression of Ctss in BMDMs treated with miR-203-3p mimic determined by RT-qPCR. (**D**) The protein expression of Ctss in BMDMs treated with miR-203-3p mimic determined by Western blot analysis (50 μg protein was loaded). (**E**) mRNA stability of Ctss following miR-203-3p mimic and miR-203-3p inhibitor evaluated using RNA degradation experiment. (**F**) The binding of miR-203-3p and Ctss detected using RIP assay. Ago2 antibody was used for immunoprecipitation of the RNA-induced silencing complex (Ago2-RISC) in BMDMs (Input: total sample before co-immunoprecipitation treatment). IgG was employed as a negative control and β-actin was used as an internal control. (**G**) Relative mRNA expression of serum Ctss detected by RT-qPCR (Relative quantification based on the expression of healthy population). (**H**) Protein expression of serum Ctss detected by ELISA. ^*^ *p* < 0.05 *vs.* healthy persons. ** *p* < 0.01. Healthy persons = 50; AS patients = 76. Statistical data were measurement data, and described as mean ± standard deviation. The paired *t* test was used for comparisons between two groups. The one-way analysis of variance was adopted for comparisons among multiple groups, followed by Tukey’s post hoc test. The experiment was repeated 3 times independently.

The TargetScan database suggested that the 206-213 site of 3'UTR region of the Ctss mRNA may be the binding site of miR-203-3p (Figure 5A). Subsequently, a dual-luciferase reporter gene assay was carried out to verify whether Ctss was a target of miR-203-3p. A marked decline in luciferase activity was observed in cells transfected with miR-203-3p mimic in combination with Ctss-wt reporter plasmid (*p* < 0.05). In contrast, co-transfection of Ctss-mut reporter plasmid and miR-203-3p mimic resulted in the complete retraction of reporter inhibition (*p* > 0.05), indicating that miR-203-3p could bind to Ctss mRNA (Figure 5B). To further verify whether Ctss was a target gene for miR-203-3p, Ctss expression was determined by conducting RT-qPCR and Western blot following transfection with miR-203-3p mimic. The results showed that the mRNA and protein expression of Ctss in BMDMs transfected with miR-203-3p mimic was significantly reduced (*p* < 0.05). The mRNA and protein expression of Ctss was increased in BMDMs upon miR-203-3p inhibitor transfection (Figure 5C, 5D; *p* < 0.05), confirming that Ctss was the target gene of miR-203-3p. In order to detect whether miR-203-3p can target Ctss and affect its stability, we carried out RNA degradation experiments. The results showed that miR-203-3p knock down slowed mRNA degradation of Ctss, while mRNA degradation of Ctss was accelerated and the stability was weakened in response to the overexpression of miR-203-3p (Figure 5E). These results further suggested that miR-203-3p can target Ctss. It is well known that miRNAs may regulate their targets by forming RNA-induced silencing complexes (RISC). In order to further explore whether miR-203-3p and Ctss co-existed in RISC complex, we used antibodies against Ago2, the key component of RISC complex, and detected the binding of Ctss and miR-203-3p using RIP assay. As shown in Figure 5F, BMDMs treated with anti-Ago2 showed increased Ctss and miR-203-3p enrichment compared to BMDMs treated with anti-IgG (*p* < 0.05). The serum levels of Ctss were also detected in AS patients, the results of which showed that serum levels of Ctss in AS patients were higher compared to those in healthy persons (Figure 5G and 5H) (*p* < 0.05). Taken together, these data indicated that Ctss was a direct target of miR-203-3p in AS.

### Ctss silencing promotes BMDM migration

**Figure 6:**
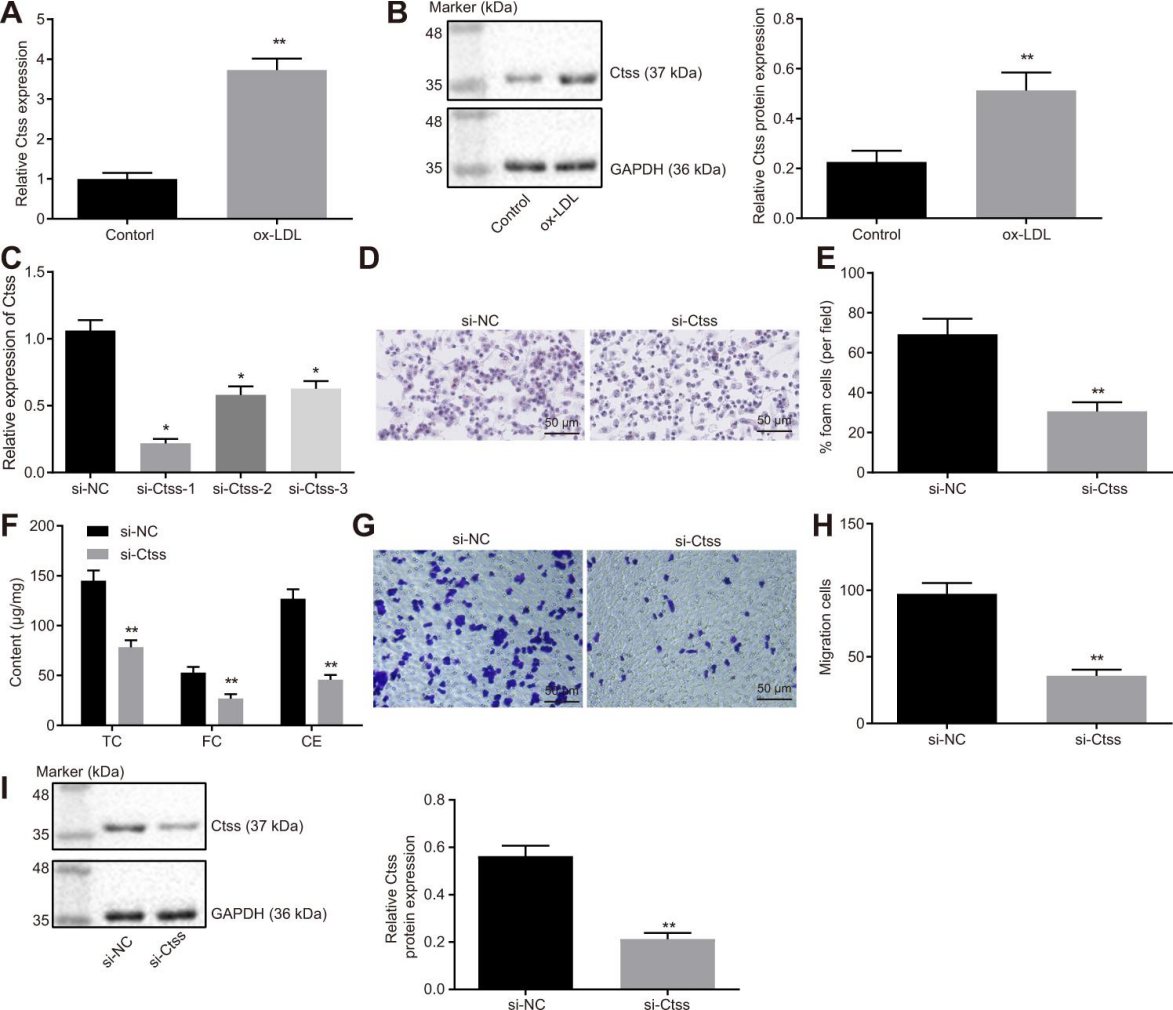
**Ctss affects BMDM expression of atherosclerotic phenotype.** (**A**) The mRNA expression of Ctss in BMDMs after 24-h treatment with ox-LDL determined by RT-qPCR. (**B**) The protein expression of Ctss in BMDMs after 24-h treatment with ox-LDL determined by Western blot analysis. (**C**) Silencing efficiency of Ctss after 24-h treatment with ox-LDL and different siRNA detected by RT-qPCR. (**D**–**E**) The proportion of foam cells in BMDMs treated with si-Ctss by oil red O staining (200 ×) (Five visual fields were randomly read and photographed, and the mean value was obtained. Each experiment was repeated three times). (**F**) Serum TC, FC and CE levels in BMDMs treated with si-Ctss determined by ELISA. (**G**, **H**) BMDM migration in response to the treatment of si-Ctss evaluated by Transwell assay (200 ×) (Five visual fields were randomly read and photographed, and the mean value was obtained. Each experiment was repeated three times). (**I**) Ctss protein expression in BMDMs in response to the treatment of si-Ctss evaluated by western blot analysis. ^**^ *p* < 0.01 *vs.* the BMDMs treated with NC mimic, si-NC or without treatment. Statistical data were measurement data, and described as mean ± standard deviation. The paired *t* test was used for comparisons between two groups. The one-way analysis of variance was adopted for comparisons among multiple groups, followed by Tukey’s post hoc test. The experiment was repeated 3 times independently.

The mRNA and protein expression of Ctss in BMDMs stimulated by ox-LDL was found to be significantly increased (Figure 6A, 6B; *p* < 0.05). All three siRNAs effectively silenced Ctss, but si-Ctss-1 has the highest silencing efficiency (Figure 6C), so si-Ctss-1 was selected for the follow-up experiments. After silencing Ctss in BMDMs, the AS-related phenotypes induced by ox-LDL were alleviated remarkably (Figure 6D–6F; *p* < 0.05). It has been previously highlighted that elevated BMDM migration can serve as an indicator of the severity of atherosclerotic plaque [19]; therefore, we measured the migration of BMDMs using Transwell assay. By using the mature BMDMs, ox-LDL (50 μg/mL) was added to the basolateral chamber of Transwell for 24-hour stimulation. The results showed that the migration of BMDMs was enhanced following Ctss silencing (Figure 6G, 6H; *p* < 0.05). Western blot analysis revealed that si-Ctss treatment decreased the protein expression of Ctss (Figure 6I; *p* < 0.05). These results indicated that BMDM migration was promoted by Ctss silencing.

### miR-203-3p transport from DCexs targets Ctss to inhibit AS-related phenotypes in BMDMs

**Figure 7:**
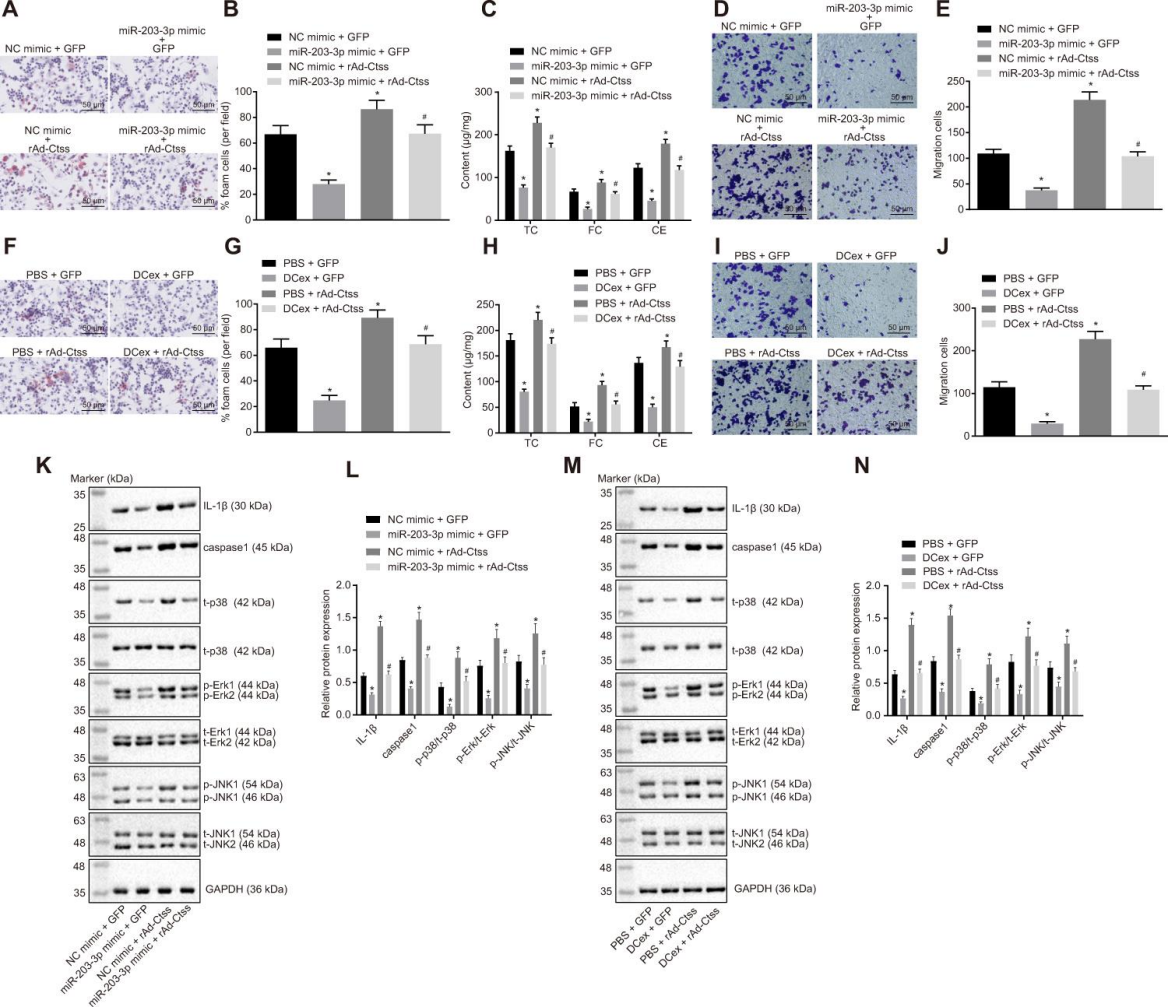
**The miR-203-3p/Ctss/p38/MAPK axis mediates the progression of AS *in vitro*.** BMDMs were treated with NC mimic or miR-203-3p in the presence of green-fluorescent protein (GFP) or rAd-Ctss. (**A**, **B**) The proportion of foam cells in BMDMs by oil red O staining (200 ×). (**C**) Serum TC, FC and CE levels determined by ELISA. (**D**, **E**) BMDM migration evaluated by Transwell assay (200 ×). BMDMs were treated with PBS or DCexs in the presence of GFP or rAd-Ctss. (**F**, **G**), The proportion of foam cells in BMDMs by oil red O staining (200 ×). (**H**) Serum TC, FC and CE levels determined by ELISA. (**I**, **J**) BMDM migration evaluated by Transwell assay (200 ×). (**K**, **L**) Protein expression of genes related to the p38/MAPK signaling pathway tested by Western blot analysis in response to the treatment of NC mimic or miR-203-3p in the presence of green-fluorescent protein (GFP) or rAd-Ctss. (**M**, **N**) Protein expression of genes related to the p38/MAPK signaling pathway tested by Western blot analysis in response to the treatment of PBS or DCexs in the presence of GFP or rAd-Ctss. The results for the inflammation-related proteins IL-1β and caspase 1 were derived from additional independent experiments. ^*^ *p* < 0.05 *vs.* the BMDMs treated with NC mimic or PBS plus GFP. ^#^ *p* < 0.05 *vs.* the BMDMs treated with miR-203-3p mimic or DCex plus GFP, or NC mimic or PBS plus rAd-Ctss. Statistical data were measurement data, and described as mean ± standard deviation. The one-way analysis of variance was adopted for comparisons among multiple groups, followed by Tukey’s post hoc test. The experiment was repeated 3 times independently.

In order to detect the target relationship between miR-203-3p in DCexs and Ctss, gain-of-function experiments were carried out by exosomes. The results of oil red O staining displayed that compared with treatment with NC mimic + GFP, the proportion of foam cells in BMDMs was decreased after treatment with miR-203-3p mimic + GFP, and was increased by treatment with NC mimic + rAd Ctss (*p* < 0.05). miR-203-3p mimic treatment in BMDMs reversed the changes of the proportion of foam cells induced by rAd Ctss (Figure 7A, 7B; *p* < 0.05). ELISA revealed that serum TC, FC and CE levels were lower following treatment with miR-203-3p mimic + GFP but were higher after treatment with NC mimic + rAd Ctss in contrast with treatment with NC mimic + GFP (*p* < 0.05). miR-203-3p mimic treatment reversed the increase of serum TC, FC and CE levels induced by rAd Ctss (Figure 7C; *p* < 0.05). Then, Transwell assay showed that BMDM migration was reduced by treatment with miR-203-3p mimic + GFP but was elevated by treatment with NC mimic + rAd Ctss in contrast to the treatment with NC mimic + GFP (*p* < 0.05). In comparison to the treatment with NC mimic + GFP, BMDM migration returned to normal level after treatment with miR-203-3p mimic + rAd Ctss (Figure 7D, 7E; *p* < 0.05). Similarly, in comparison to the treatment with PBS + GFP, the proportion of foam cells, serum TC, FC and CE levels and migration were all diminished following treatment with DCexs + GFP, and enhanced after treatment with PBS + rAd Ctss; these factors returned to normal levels after treatment with DCexs + rAd Ctss (Figure 7F–7J; *p* < 0.05). These results suggested that miR-203-3p mimic and DCexs individually inhibited AS-related phenotypes, while over-expression of Ctss intensified AS-related phenotypes. Furthermore, miR-203-3p mimic or DCexs reversed the augmentation of AS phenotype induced by over-expression of Ctss.

In addition, western blot analysis exhibited that miR-203-3p mimic decreased expression of p-p38/t-p38, p-JNK/t-JNK, p-ERK/t-ERK and inflammation-related genes (IL-1β and caspase 1) (*p* < 0.05). Treatment with rAd Ctss elevated expression of p-p38/t-p38, p-JNK/t-JNK, p-ERK/t-ERK and inflammation-related genes (IL-1β and caspase 1), which was neutralized by miR-203-3p mimic (Figure 7K, 7L; *p* < 0.05). Similarly, expression of p-p38/t-p38, p-JNK/t-JNK, p-ERK/t-ERK and inflammation-related genes (IL-1β and caspase 1) was reduced following treatment with DCexs (*p* < 0.05). The expression of p-p38/t-p38, p-JNK/t-JNK, p-ERK/t-ERK and inflammation-related genes (IL-1β and caspase 1) was enhanced by treatment with Ctss overexpression, which was normalized by DCexs (Figure 7M, 7N; *p* < 0.05). Collectively, these results indicated that miR-203-3p in DCexs repressed AS-related phenotypes in BMDMs through the Ctss-dependent p38/MAPK signaling pathway.

### miR-203-3p mediated the effects of DCexs *in vivo*

**Figure 8:**
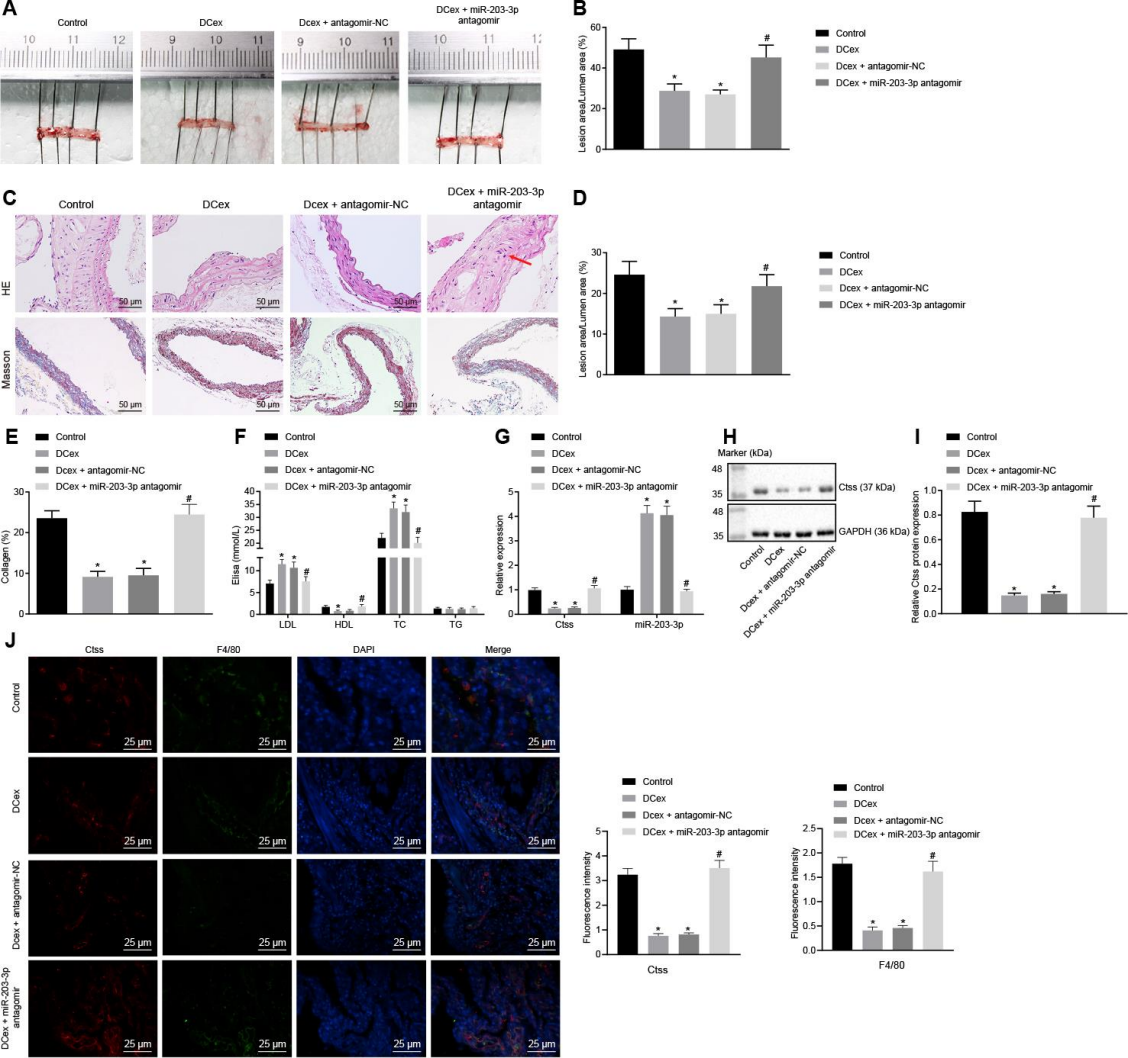
**DCex and miR-203-3p contribute to alleviate AS *in vivo*.** The HFD-fed ApoE^-/-^ mice did not receive any treatment as controls, or were treated with DCex alone, DCex + antagomir-NC, or miR-203-3p antagomir (n = 8 for each group). (**A**, **B**) Oil red O staining for atherosclerotic plaque in ApoE^-/-^ mice. (**C**–**E**) HE staining (200 ×) and Masson staining (200 ×) for atherosclerotic plaque in ApoE^-/-^ mice (In panel C, red arrows show plaque location). (**F**) Serum LDL, HDL, TC, and TG levels in ApoE^-/-^ mice determined by ELISA. (**G**) miR-203-3p expression and mRNA expression of Ctss in vascular tissues in ApoE^-/-^ mice evaluated by RT-qPCR. (**H**, **I**) The protein expression of Ctss in vascular tissues in ApoE^-/-^ mice evaluated by Western blot analysis. (**J**) Immunofluorescent double staining for the expression of F4/80 and Ctss (400 ×). ^*^ *p* < 0.05 *vs.* mice without treatment; ^#^ *p* < 0.05 *vs.* mice treated with DCex. Statistical data were measurement data, and described as mean ± standard deviation. The one-way analysis of variance was adopted for comparisons among multiple groups, followed by Tukey’s post hoc test. The experiment was repeated 3 times independently.

Finally, in order to validate the effect of miR-203-3p on the development of AS in mice, HFD-fed ApoE^-/-^ mice were injected with PBS as control, injected with mature DCex containing miR-203-3p, or injected with DCex +miR-203-3p antagomir or DCex + antagomir-NC to validate the effect of miR-203-3p *in vivo*. HFD ApoE^-/-^ mice treated with PBS or DCex + miR-203-3p antagomir presented with poor mental states, decreased activity, and a few of them exhibited symptoms of abdominal distension and diarrhea. The DCex-treated mice presented with good mental states, lustrous hair, sensitive reaction and normal food intake. After 12 weeks, the mice were euthanized according to the aforementioned method for further experimental examination. Oil red O staining and HE stain were conducted, and the size of the plaques was observed in order to detect atherosclerotic plaque lesions in mice. The results showed that the plaque size of artery in HFD-fed mice treated with DCex or DCex + antagomir-NC was significantly smaller than that in HFD-fed mice treated with PBS or DCex + miR-203-3p antagomir. Intimal thickening and fibrous cap formation were observed in HFD-fed mice treated with PBS or DCex + miR-203-3p antagomir, with varying degrees of uneven thickness of the aortic wall and obvious atherosclerotic plaques (Figure 8A–8E; *p* < 0.05). Additionally, serum TC, TG, high-density lipoprotein (HDL) and LDL levels were detected using ELISA, which revealed that the levels of TC and LDL in the HFD-fed mice treated with DCex or DCex + antagomir-NC were elevated, while those of HDL were reduced versus HFD-fed mice treated with PBS or DCex + miR-203-3p antagomir (Figure 8F; *p* < 0.05). The expression of Ctss and miR-203-3p detected by RT-qPCR showed that Ctss was significantly increased, while miR-203-3p expression was markedly decreased in HFD-fed mice treated with PBS or DCex + miR-203-3p antagomir (Figure 8G; *p* < 0.05), which was in accordance with the protein expression of Ctss assessed using Western blot analysis (Figure 8H–8I; *p* < 0.05). Next, in order to evaluate whether miR-203-3p actually influenced the expression of Ctss in atherosclerotic plaque in ApoE^-/-^ mice, sections of aortic sinuses of mice were examined by immunofluorescence double staining. BMDMs in plaques were labeled with antibody against F4/80 (green), and Ctss in plaques were labeled with antibody against Ctss (red) with DAPI (blue) indicating nucleus (Figure 8J). Relative quantification of Ctss and BMDMs could be observed from the image: compared with the control treatment, Ctss were significantly decreased after DCex or DCex + antagomir-NC treatment, and returned to normal levels following the treatment with DCexs + miR-203-3p antagomir (*p* < 0.05). In each group, the Ctss-positive areas were mainly located in the aortic root, while the F4/80-labeled BMDMs were highly coincident with the Ctss-positive areas and AS lesion areas. The above results suggest that DCexs-derived miR-203-3p could reduce the progression of AS.

## DISCUSSION

In the early stages of atherosclerotic plaque formation, the function of macrophages is to firstly degrade lipoproteins and induce inflammatory responses, and its second role includes facilitating cell apoptosis and impeding the growth of plaque. Interestingly, the differentiation of macrophages can be regulated by miRNAs, due to their ability to modulate the activities of key transcription factors during atherosclerotic plaque formation [20]. miR-214-3p has been highlighted to play a critical role in the pathogenesis of AS, whereas miR-126-5p prevents atherosclerotic lesion formation by promoting the proliferation of arterial ECs [21, 22], and the mechanisms of action includes binding to different genes. Typically, these functional miRNAs are shipped by exosomes secreted by monocytes in the blood stream. Zhou et al. demonstrated that miR-203 could be transferred to other cells via exosomes [23]. The present study provides evidence that DCexs containing miR-203-3p could alleviate AS progression through the inhibition of Ctss, suggesting a novel target that can minimize the risks of AS development in the patient population.

Our findings revealed that miR-203-3p was significantly diminished in the serum of AS patients, whereas Ctss was markedly up-regulated in BMDMs challenged by ox-LDL. Initially, the AS-related expression profile microarray GSE56143 was retrieved, which revealed a high expression of Ctss in AS patients. Moreover, several artery diseases have been demonstrated to have an aberrant expression of Ctss. In addition, Qin et al*.* illustrated that Ctss expression was profoundly elevated in abdominal aortic aneurysm (AAA) lesions of mice models, and that the incidence of AAA in ApoE^–/–^ Ctss^–/–^ mice (10%) was significantly lower compared to ApoE^–/–^ Ctss^+/+^ mice (80%) [24]. Furthermore, minimal or no expression of Ctss was detected in normal human arteries, while macrophages of atherosclerotic lesions were observed with an abundant expression of Ctss [25]. All of these findings are highly indicative of the close association that exists between Ctss and blood vessel diseases. To further study the mechanism of Ctss in AS, regulatory miRNAs of Ctss were predicted with the use of RAID and DIANA TOOLS, and the common results identified miR-203-3p as the candidate miRNA, which was further identified by a dual luciferase reporter gene assay. Similarly, significantly reduced expression of miR-203 has been found in aortic atherosclerotic plaques of endogenous high Ang II ApoE^–/–^ mice relative to that sham-operated mouse [11]. Another study also found that osteogenesis was inhibited by up-regulation of miR-203-3p and was promoted upon miR-203-3p silencing, highlighting the involvement of miR-203-3p in the suppression of osteogenesis [26]. These findings indicated that dysregulation of miR-203-3p serves as an indicator of several diseases. The current study also examined the role of miR-203-3p in DCexs and Ctss in mice with AS, the results of which revealed that in addition to significantly alleviating AS progression, miR-203-3p restoration can also rescue Ctss-induced aggravation of AS-related phenotypes. This finding came about following the revelation that miR-203-3p restoration reduced atherosclerotic plaque sizes, foam cell formation, as well as TC, FC and CE serum levels. Further accumulation of FC and CE in macrophage-derived foam cells, leads to the formation of atherosclerotic plaque [27]. Specifically, cysteinyl cathepsins are also involved in the degradation of HDL, thereby diminishing macrophage-derived foam cell cholesterol efflux [28]. Contrariwise, the suppression of Ctss was previously revealed to enhance both HDL and cholesterol efflux from peritoneal macrophages [29].

In addition, our study found that miR-203-3p was delivered by the exosomes secreted by DCs into BMDMs, resulting in the suppression of AS progression. DCs in tissues usually begin to migrate through lymphatics into T cell-enriched regions of lymph nodes in response to pathogens like out-of-body microbes or abnormal intracorporal molecules, and undergo maturation by elevating the levels of T-cell costimulatory molecules, such as CD80/B7-1, CD83, and CD86/B7-2 [30]. Conventional DCs also exert a dominant role in the atherosclerotic process as they are directly engaged in cholesterol homeostasis and immune responses [31], which was consistent with our finding that mature DCs could lower the levels of TC, FC and CE. Moreover, DCexs not only act as stable vesicles with long shelf-lives, but their immune stimulatory characteristics are easily manipulated, making DCexs promising immunotherapeutic targets [32]. Additionally, Figueiredo et al. validated our finding that selective Ctss inhibition markedly decreases the size of atherosclerotic plaques [33]. Moreover, our study provided evidence that miR-203-3p inhibited the progression of AS through the p38/MAPK signaling pathway. As previously described, miR-203 upregulation resulted in suppressed c-Jun, p38 and MAPK in hepatocellular carcinoma cells [34]. Interestingly, enhanced cholesterol levels induced by exposure to LDL-activated the p38/MAPK signaling pathway elevated the ratio of CE to FC, whereas blocking p38/MAPK with SB203580 or siRNA decreased the intracellular accretion of FC and CE in macrophages [35]. These mechanisms support the hypothesis that the miR-203-3p/Ctss/p38/MAPK axis might modulate AS progression; however, its role in AS requires further investigations.

**Figure 9:**
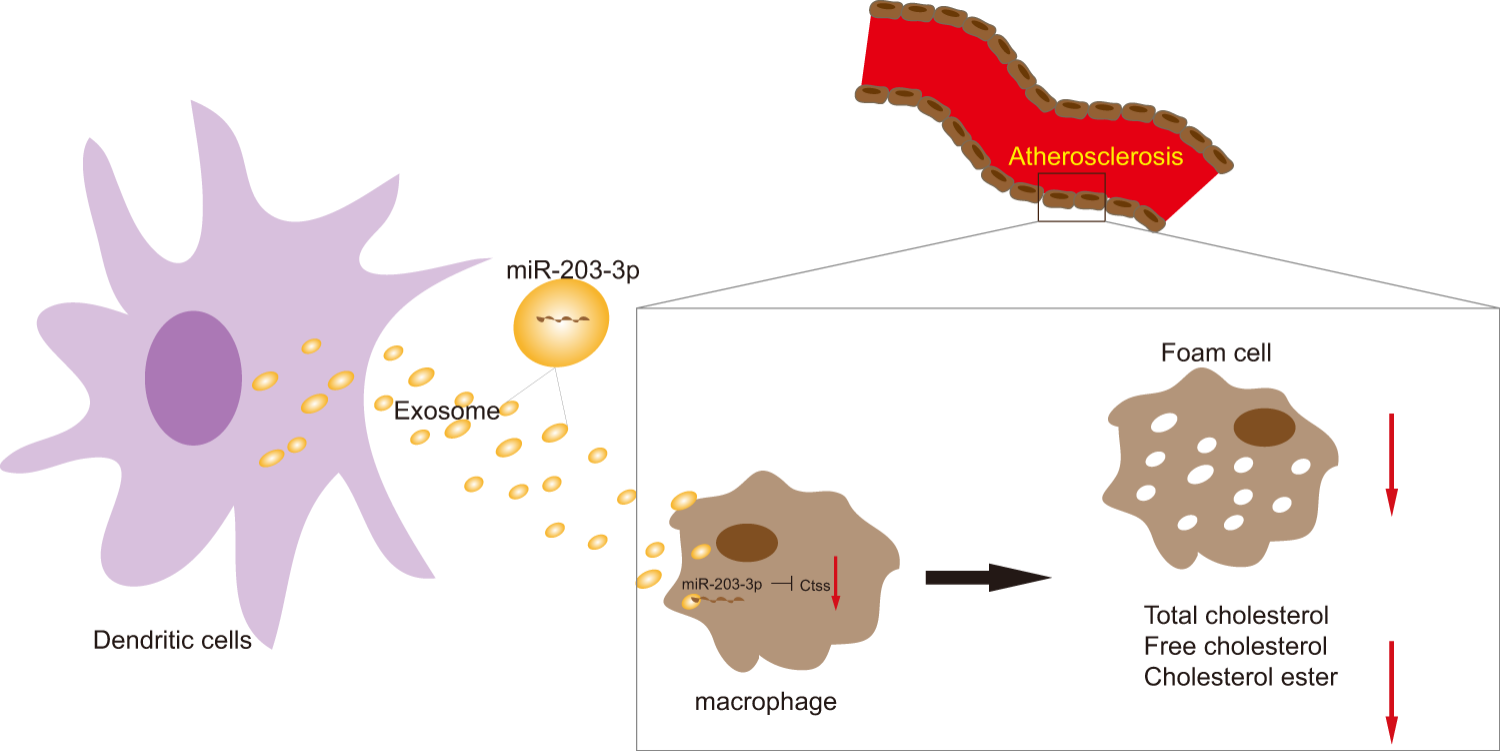
The mechanism diagram shows that transfer of exosomal miR-203-3p from dendritic cells to BMDMs reduces BMDM-derived foam cells and the levels of TC, FC and CE by impairing Ctss expression, thus suppressing the development of atherosclerosis.

In conclusion, these findings highlighted the impact of DCexs-derived miR-203-3p on AS by providing evidence at cellular and mouse levels (Figure 9). This study not only clarifies DCexs-derived miR-203-3p as a promising target for AS treatment but also provides insight into the potential of exosomal miRNA-based therapy. Nevertheless, it was reported that exosomes encompass numerous bioactive molecules, including proteins and miRNAs, of which miRNAs occupy a large proportion [36]. Therefore, although this study focused on miR-203-3p, other miRNAs may also contribute to the DCexs-induced protective effects on AS. Because the exosomes of all other miRNAs were not depleted in the present study, we cannot rule out the possibility that additional miRNAs are also equally attributed to the repressive effects of DCexs on AS. Further studies are needed to clarify the function of other miRNAs in exosomes in AS. Ongoing efforts that combine delineation of underlying molecular mechanisms involving the p38/MAPK signaling pathway will provide innovative insights into the optimal management of AS in patients.

## MATERIALS AND METHODS

### Ethics statement

Signed informed consents were obtained from all participating patients. All protocols conducted in the present study were approved by the ethics committee of the Yulin First People’s Hospital. All animal experiments were carried out in strict accordance with the Guide for the Care and Use of Laboratory Animals of the National Institutes of Health. All efforts were made to minimize animal number and suffering.

### miRNA and mRNA expression profiles in AS

AS-related expression profile microarray GSE56143 was retrieved from the Gene Expression Omnibus (GEO) database (https://www.ncbi.nlm.nih.gov/geo/). The microarray was comprised of three control samples and three atherosclerosis model samples. The sequencing platform was GPL6887 (Illumina MouseWG-6 v2.0 expression beadchip). The deferentially expressed genes (DEGs) were then analyzed using the R language with the screening threshold set at |log foldchange| > 2, and *p* value < 0.05.

### Information of patients

From January 2016 - April 2017, 76 patients with AS (calculated mean age: 51.88 ± 7.24 years) and 49 healthy volunteers (calculated mean age: 49.24 ± 9.05 years) from the Yulin First People’s Hospital were enrolled in the current study. Peripheral blood samples were collected from patients and controls from 8 - 10 a.m. by vacuum vascularization. After a 15-minute centrifugation, the plasma was divided into 4 equal parts and stored in a refrigerator at -80° C for later use. Pathological diagnosis was made in accordance with the World Health Organization criteria. The exclusion criteria included patients with Takayasu arteritis, aneurysms, acute and chronic infections, immune reactive diseases, hematological diseases, recent trauma or surgical history. The clinical characteristics of volunteer and patients are shown in Table 1.

**Table 1:** The clinical characteristics of volunteer and patients.

	**Volunteer (n = 49)**	**Patients (n = 76)**
Age	49.24 ± 9.05	51.88 ± 7.24
Gender (male: female)	22: 27	44: 32
Blood glucose (mmol/L)	10.21 ± 1.43	11.83 ± 1.92
HDL-C (mmol/L)	1.39 ± 0.28	1.42 ± 0.36
LDL-C (mmol/L)	2.73 ± 0.52	3.15 ± 0.69
Hb (mmol/L)	106.3 ± 14.63	131.8 ± 9.21
Systolic pressure (mmHg)	116.2 ± 11.3	134.25 ± 14.85
Diastolic pressure (mmHg)	75.14 ± 10.60	79.14 ± 8.96

Note: HDL-C, high density lipoprotein cholesterol; LDL-C, low-density lipoprotein cholesterol.

### Quantification of lipid components in the blood

Patients’ serum or cell culture supernatant was collected, and total cholesterol (TC), low density lipoprotein (LDL), triglyceride (TG) and Ctss levels of human and mice were determined according to the instructions of enzyme-linked immunosorbent assay (ELISA) kits (TW041962/tw035400, TWp026709/tw040516, TWp026468/tw040594, TW reagent, Shanghai, China). The optical density (OD) values of each well were measured at a wavelength of 450 nm with a microplate reader (BS-1101, Nanjing DeTie Laboratory Equipment Co., Ltd., Nanjing, Jiangsu, China) with blank wells serving as negative controls. The levels of TC and free cholesterol (FC) were measured respectively, and the difference between the two values was determined to be the cholesterol esters (CE) level.

### Extraction and culture of bone marrow-derived macrophages (BMDMs) in mice

The C57BL/6 mice were euthanized under excessive anesthesia. The femur and the tibia on both legs were removed with scissors, and the bone marrow was washed out with a 1 mL syringe filled with cell culture medium, collected and triturated repeatedly in a petri dish until the bone marrow was dispersed evenly. The bone marrow cell suspension was then seeded into Petri dishes or culture plates, and the culture medium for BMDMs was renewed every 2-3 days. Next, the cells were rinsed with buffered saline (PBS) before being added to the culture medium. After 7 days of culture, the BMDMs were differentiated and were deemed suitable for further experimentation. BMDMs were transformed into foam cells by incubation for 48 hours with 50 μg/mL oxidized (ox)-LDL (Union-Biology Co., Ltd, Beijing, China) in serum-free Roswell Park Memorial Institute (RPMI)1640 medium containing 0.3% bovine serum albumin (BSA). The cells were then assigned into the control group (the same volume of PBS solution) and Ox-LDL intervention group.

### Extraction and identification of DCexs

C57BL/6 male mice (aged 6 - 8 weeks) were used in the experiments. The femurs of the mice were soaked in 75% ethanol for 2 min, and rinsed twice with complete RPMI 1640 medium, after which the ends of the femur were cut open. After the femur was rinsed with 5 mL complete RPMI 1640 medium, the cells were transferred into 15 mL centrifuge tubes. After centrifugation, the cells were counted and resuspension was carried out with RPMI 1640 containing 20 ng mL granulocyte-macrophage colony stimulating factor (GM-CSF) and supplemented with 100 U/mL penicillium and 100 U/mL streptomycin. BMDMs were cultured in 10 cm plates at the density of 2 × 10^6^ cells each well with 5% CO_2_ and saturated humidity at 37° C. The culture medium was collected and centrifugation was carried out at 4° C at 100000 g overnight for the removal of the DCexs in the medium [37]. Next, the DCs received treatment with vitamin D3 (10 nM) or lipopolysaccharide (LPS; 200 ng/mL) for 24 hours. The calcium carrier ionomycin (100 nM; HY-13434, MCE, Shanghai, China) was added one hour before the end of the experiment [6].

The cells were seeded in RPMI 1640 medium containing 10% fetal bovine serum (FBS) at a density of 2 × 10^6^ cells per well, followed by incubation at 37° C with 5% CO_2_ in air. Two hours after the removal of non-adherent cells, the culture medium was replaced with medium containing recombinant human GM, 1000 U/mL CSF and 500 U/mL IL4. Half of the culturing medium was then renewed every 48 hours. On day 5, tumor necrosis factor (TNF; 1000 U/mL) was added to promote the maturation of DCs. After 48 h, DCs were lightly triturated, and the cultured supernatant was collected after 24 h stimulation with human LPS (10 ng/mL). Large cell fragments were removed by centrifugation at 900 × g for 10 min and smaller fragments were removed by filtration through a 0.22 μm membrane. After a 100000 × g ultra-centrifugation for 1 hour, the supernatant was removed, following resuspension with PBS and another ultra-centrifugation. The DCexs were collected, identified under a transmission electron microscope and stored at about 80° C for later use. The exosome size was measured with a NanoSight’s instrument (Nanosight, Marlvern, UK) [38]. The surface markers of exosomes were identified by Western blot analysis. The exosome suspension was concentrated, and its protein expression was determined using a bicinchoninic acid (BCA) kit (23227, Thermo Fisher Scientific Inc., Waltham, MA, USA). The protein was denatured, separated by sodium dodecyl sulfate polyacrylamide gel electrophoresis (SDS-PAGE) and transferred into membranes. Finally, the expression of exosomes-specific labeled proteins lysosomal associated membrane protein 1 (LAMP1), LAMP2 and CD63 was measured.

### Cell treatment

The BMDMs were treated with plasmids of negative control (NC) mimic plasmid, miR-203-3p mimic plasmid, small interference (si)-NC plasmid, si-Ctss plasmid, plasmid of NC mimic + green-fluorescent protein (GFP), plasmid of miR-203-3p mimic + GFP, plasmid of NC mimic + rAD-Ctss, plasmid of miR-203-3p mimic + rAD-Ctss, DCex-NC-inhibitor or DCex-miR-203-3p-inhibitor. After 24-h stimulation in 50 mg/L ox-LDL or 8-h incubation with 10 μm GW4869, the BMDMs were treated with the corresponding mimics and inhibitors of miR-203-3p or Ctss [39, 40].

Next, the DCs were treated with DCex-NC-inhibitor, DCex- miR-203-3p -inhibitor, PBS + GFP, DCex + GFP, PBS + rAD-Ctss or DCex + rAD-Ctss, and with PBS + dimethyl sulfoxide (DMSO), PBS + GW4869, DCex + DMSO (culture supernatant of DCs treated with DMSO) or DCex + GW4869 (culture supernatant of DCs treated with GW4869).

Mimic and inhibitor of miR-203-3p and rAD-Ctss were purchased from Dharmacon (Lafayette, CO, USA). Cell density was adjusted according to the cell growth rate and laid on 6-well plates to make the cell confluence reach 80% - 90% on the 2nd day of transfection. The cells were transfected using a lipofectamine 2000 kit (Invitrogen Inc., Carlsbad, CA, USA). The transfection steps were carried out according to the instructions. After 8 h of transfection, fresh culture medium was changed, and the follow-up experiments were carried out after 48 h.

### Dual luciferase reporter gene assay

The binding sites between Ctss and miR-203-3p were predicted using an online prediction tool. Subsequently, a dual luciferase reporter gene assay was used for the verification of the target relationship between Ctss and miR-203-3p. Next, Ctss mutant (pGL3-Ctss-mut) and wild type (pGL3-Ctss-wt) plasmids were constructed. The reporter plasmids were co-transfected into HEK293 cells with over-expressed miR-203-3p plasmid and pRL-TK (internal reference plasmids expressing renilla luciferase), respectively. After transfection for 24 hours, the cells were lysed in accordance with the instructions of the TransDetect Double-Luciferase Reporter Assay Kit (FR201-01, Transgene Biotech, Beijing, China), after which the supernatant was collected. The luciferase activity was then tested with a dual-luciferase ^®^Reporter Assay System (E1910, Promega Corporation, Madison, WI, USA). A total of 100 μL luciferase reaction reagent (equilibrium to room temperature) was added to the test tube, which was followed up by the addition of 20 μL cell lysate and gently mixed to determine the firefly luciferase signals. Then 100 μL luciferase reaction reagent II (equilibrium to room temperature) was added to determine the renilla luciferase. The ratio of firefly luciferase to renilla luciferase (FL/RL) was defined as relative luciferase activity.

### RNA binding protein immunoprecipitation (RIP) assay

The cultured cells were rinsed twice with cold PBS, added with 10 mL PBS, scraped off with a cell scraper and aspirated into a centrifuge tube. The cells then underwent centrifugation at 403 × g for 5 min at 4° C, and the supernatant was discarded. Then, the cells were added with RIP lysis buffer (N653-100 mL, Shanghai Haoran Biotechnology Co., Ltd., Shanghai, China), triturated thoroughly, mixed and lysed on ice for 5 min to prepare the cell lysates. Next, 50 μL magnetic beads were added in each tube with addition of 0.5 mL RIP wash buffer (EHJ-BVIS08102, Xiamen Huijia Biotechnology Co., Ltd., Xiamen, Fujian, China). The tubes were briefly whirled and placed on a magnetic separator for bead aggregation, after which the supernatant was discarded, with the beads retained. The beads were washed again, and each tube was added with 100 μL RIP Wash Buffer to re-suspend the beads, and added with 5 μg argonaute 2 (Ago2) antibody (P10502500, Shenzhen Otwo Biotechnology Co., Ltd., Shenzhen, China). The normal mouse immunoglobulin G (IgG) was regarded as the NC and incubation was carried out for 30 min at room temperature with the supernatant discarded. Then the magnetic beads were rinsed twice with 0.5 mL RIP wash buffer for subsequent experiments. Afterward, 900 μL of RIP Immunoprecipitation Buffer (P10403138, Shenzhen Otwo Biotechnology Co., Ltd., Shenzhen, China) was separately added to the magnetic bead-antibody mixture, and the cell lysate was centrifuged at 35068 × g for 10 min at 4° C. The supernatant was aspirated to a new eppendorf (EP) tube (LBCT015S, Beijing Beifangtongzheng Biotechnology Development Co., Ltd., Beijing, China), and 100 μL of supernatant was aspirated into the magnetic bead-antibody tube with 1.0 mL as the final volume of the immunoprecipitation reaction. Next, the tubes were incubated overnight at 4° C, and the magnetic beads were rinsed 6 times with 0.5 mL RIP wash buffer, and finally the tubes were incubated with 150 μL of proteinase K buffer for 30 min at 55° C for RNA purification. Total RNA was extracted using the conventional TRIZOL method, and detected by RNA isolation and quantitation.

### RNA degradation experiment

Cells were planted in 12-well plates. The next day, following adherence, the cells were transfected with miR-203-3p mimic, miR-203-3p inhibitor and corresponding negative control. After 12 hours, the cells were treated with 8 μM actinomycin D for 0 hour, 2 hours and 4 hours. RT-qPCR was performed to detect the expression of Ctss mRNA, and three replicates were set in each group. Each transfection group received treatment with actinomycin D for 0 hour as the control group and normalization was carried out for line graph.

### RNA isolation and quantification

Total RNA was extracted from cells and tissues and exocrine RNA using the RNA extraction kit (AM1552, Thermo Fisher Scientific Inc., Waltham, MA, USA) in accordance with the instructions. Next, the concentration of RNA was determined by RT-qPCR. The primers used in this study were synthesized by Takara Biotechnology (Dalian, Liaoning, China) (Table 2). Synthesis of complementary DNA (cDNA) was performed according to the one-step miRNA reverse transcription kit (D1801, HaiGene Co., Ltd., Harbin, Heilongjiang, China). The reaction for the amplification system was 10 μL with 1 μL transcribed cDNA on a quantitative PCR (AQ101-02, Transgene Biotech, Beijing, China). A fluorescent quantitative polymerase chain reaction (PCR; ABI ViiA 7, DAAN Gene Co., Ltd. of Sun Yat-sen University, Guangzhou, Guangdong, China) was carried out for quantification. The serum samples were then added with *C. elegans* miR-39, which served as the internal control for normalization of sample-to-sample variation. U6 small nuclear RNA was used as an internal control for cell samples, and glyceraldehyde-3-phosphate dehydrogenase (GAPDH) was used as an internal control for Ctss. The fold changes were calculated using the relative quantification (the 2^-ΔΔCt^ method): ΔΔCt = ΔCt _experimental group_ - ΔCt _control group_, ΔCt = CT _(target gene)_ - CT _(internal reference)_ [41].

**Table 2:** Primer sequences for RT-qPCR.

**Gene**	**Primer sequence (5'-3')**
Ctss	F: GCCTGATTCTGTGGACTGG
	R: GATGTACTGGAAAGCCGTTGT
miR-203-3p	F: ACACTCCAGCTGGGGTGAAATGTTTAGGACCA
	R: CTCAACTGGTGTCGTGGA
GAPDH	F: GCAAATTCCATGGCACCGT
	R: TCGCCCCACTTGATTTTGG
U6	F: CTCGCTTCGGCAGCACAT
	R: AACGCTTCACGAATTTGCGT
ß-Actin	F: CGTTGACATCCGTAAAGACC
	R: AACAGTCCGCCTAGAAGCAC

Note: RT-qPCR, reverse transcription quantitative polymerase chain reaction; Ctss, cathepsin S; miR-203-3p, microRNA-203-3p; GAPDH, glyceraldehyde-3-phosphate dehydrogenase.

### Western blot analysis

A radio-immunoprecipitation assay (RIPA) lysis buffer (R0010, Beijing Solabio Life Sciences Co., Ltd., Beijing, China) was employed to extract the total protein in tissues, cells or exosomes in strict accordance with the instructions. The cells were lysed at 4° C for 15 minutes and centrifugation was carried out at 25764 ×g for 15 minutes. The protein concentration of each sample was determined and quantified using a BCA kit (20201ES76, Yeasen Biotechnology Co., Ltd., Shanghai, China). The 50 μg proteins were separated by SDS-PAGE, and transferred onto a polyvinylidene fluoride (PVDF) membrane using the wet-transfer method. The membranes were then blocked with 5% skim milk for 1 hour at room temperature and incubated with diluted primary antibody, rabbit antibodies to CD63 (1:1000, ab68418), CD83 (1:1000, ab172350), CD80 (1:500, ab254579), CD86 (1:1000, ab112490), LAMP1 (1:1000, ab24170), LAMP2 (1:1000, ab203224), Ctss (1:1000, ab232740), total- (t-) p38 (1:1000, ab170099), phosphorylated (p)-p38 (1:1000, ab47363), p-JNK (1:1000, ab124956), t-JNK (1:1000, ab179461), and mouse antibodies to t-ERK (1:1000, ab184699), p-ERK (1:5000, ab50011), interleukin 1 beta (IL-1β; 1:1000, ab9722), caspase 1 (1:1000, ab207802) and GAPDH (dilution ratio of 1:5000, ab8245) at 4° C overnight. After three rinses (5 minutes each time) with tris Buffered Saline with Tween (TBST) rinses, the membrane was incubated overnight with horseradish peroxidase (HRP)-conjugated goat anti-rabbit (dilution ratio of 1:20000, ab205718) or goat anti-mouse antibody (dilution ratio of 1:5000, ab6789) for 1 h at room temperature. All aforementioned antibodies were purchased from Abcam Inc. (Cambridge, UK). Next, the immunocomplexes on the membrane were visualized using enhanced chemiluminescence (ECL) reagent and band intensities were quantified using ImageJ 1.48u software (National Institutes of Health, Bethesda, MD, USA). The ratio of the gray value of the target band to GAPDH was representative of the relative protein expression.

### Transwell assay

The apical chamber of the bottom membrane of Transwell chamber was coated with Matrigel from BD company (Franklin Lakes, NJ, USA). Matrigel was polymerized into gel at 37° C for 30 minutes, and the basement membrane was hydrated prior to being used. Cells were seeded into apical chamber of a 24-well transwell chamber (8 μm) with 200 μL fresh medium at a density 5 × 10 cells/well, while the corresponding cells or 500 μL culture medium containing 10% FBS was placed in the basolateral chamber. Three parallel wells were set for each group. A cotton swab was used for the removal of the non-invaded cells after 24 hours of incubation at 37° C with 5% CO_2_ in air and cells that invaded to the lower membrane of the chamber were fixed using 4% paraformaldehyde solution at 4° C, and then stained with crystal violet (C0121, Shanghai Beyotime Biotechnology Co., Ltd., Shanghai, China) for 20 minutes. The invasive cells stained in purple were photographed and counted in 5 random fields under an inverted fluorescence microscope (TE2000, Nikon, China). The mean value was the number of cells crossing the chamber in each group. Each experiment was conducted in triplicates.

### Establishment of AS animal models

C57BL/6 pure male ApoE^-/-^ mice (Model Animal Research Center of Nanjing University, Nanjing, Jiangsu, China) were kept in a specific-pathogen-free (SPF) level laminar stream shelf of a barrier system with regular ultraviolet irradiation. The mouse cage, cushion, drinking water and diet were sterilized under high pressure. The relative humidity ranged from 40% - 60% with room temperature of 24° C - 26° C. Animals (about 8-weeks old) were then fed a high-fat (HFD) diet, comprising of 15% fat and 0.35% cholesterol for 12 weeks. Mice fed with HFD diet received treatment with PBS (as control), DCexs alone (20 μg DCexs in 100 μL PBS, injected once every week through the caudal vein), Dcex + antagomir-NC (100 nM, injected once every other day through the caudal vein), or DCexs + miR-203-3p antagomir (100 nM, injected once every other day through the caudal vein) (n = 8 for each group) [39].

On the 12^th^ week after the experiment, the mice were euthanized with 3% pentobarbital sodium (P3761, Sigma-Aldrich Chemical Company, St Louis, MO, USA). With the limbs fixed, the chest and abdomen were incised open along the midline to separate the muscles and subcutaneous tissues. The heart and aorta were exposed to collect blood samples from the heart with a 1 mL syringe. Subsequently, the aorta was dissected along the longitudinal axis, and 2-3 cm samples from the heart to the root of the aorta were sectioned, stained with Hematoxylin-eosin (HE), Masson staining and immunohistochemical staining. Subsequently, the area of AS lesions was calculated on each surface. The size of the plaque was expressed as a ratio of the plaque to the total aortic surface. The serum and plasma were stored at -20° C after centrifugation.

### HE staining

The paraffin-embedded sections were dewaxed, and the nucleus was stained with hematoxylin for 5 minutes, differentiated by 1% hydrochloric acid for 30 seconds, and finally observed under a microscope. After cytoplasm staining by eosin for 15 minutes, the sections were reacted with 80%, 95% ethanol I, 95% ethanol II, and then soaked for 5 minutes in 100% ethanol I, 10 minutes in 100% II ethanol, and then soaked twice in xylene (10 minutes each time). Finally, the sections were sealed with neutral gum and observed under an optical microscope.

### Oil red O staining

The full-length aorta was rinsed for 30 minutes. After the cautious removal of the peripheral fat and muscle tissues, the blood vessels were dissected, and soaked in distilled water for 30 minutes. Oil red O working solution was prepared with 100 mL distilled water, mixed and allowed to stand for 10 min, after which it was filtered and used for staining for 0.5 - 1 hour. The samples were differentiated with 70% ethanol until the plaques were red and the non-plaque regions were white and transparent. The samples were infiltrated with distilled water, carefully flattened on the cover glass and photographed.

The BMDMs of mice were cultured in a cell plate and stained with oil red O after receiving the corresponding stimulation (ox-LDL stimulation for 24 hours). Afterwards, the BMDMs in each well were fixed with 50% isopropanol solution for 1 minute and stained with freshly-prepared oil red O working solution for 10 - 15 minutes. The excess staining solution was removed with 60% ethanol for 1 min. The cells were then photographed under a microscope and the results were analyzed. Five visual fields were randomly read and photographed, and the mean value was obtained. Each experiment was conducted in triplicates.

### Masson staining

Masson staining was carried out according to the Masson trichromatic staining kit (Nanjing SenBeiJia Biological Technology Co., Ltd., Nanjing, Jiangsu, China). The Masson complex staining solution (solution A) was added to the sections to stain for 5 minutes, followed by phosphomolybdic acid reagent (solution C) staining for 5 minutes and aniline blue reagent (solution D) staining for 5 minutes. Finally, the sections were differentiated twice with the differentiation solution (solution B) (30 seconds - 60 seconds each time), dehydrated with 95% alcohol, anhydrous alcohol, then cleared and sealed.

### Immunofluorescence

Frozen sections were rewarmed at room temperature, fixed with ice-acetone and air-dried. The sections were then placed in an antigen repair box and soaked with 0.3% triton X-100 at room temperature to allow the rupture of cell membranes. Subsequently, the sections were sealed with 5% BSA at room temperature, and probed overnight at 4° C with the primary antibody, rabbit antibody to Ctss (ab232740, 1:200) and rat antibody to F4/80 (ab6640, 1:200). The secondary antibody, donkey anti-rabbit IgG (ab150075, 1:200, Abcam Inc., Cambridge, UK) or goat anti-rat antibody to IgG (ab150157, 1:200, Abcam Inc., Cambridge, UK) was added for incubation at room temperature for 3 h under conditions void of light. Next, the sections were counter-stained with 4',6-diamidino-2-phenylindole (DAPI), sealed with anti-fluorescence quenching sealing solution, observed and photographed under a fluorescence confocal microscope.

### Statistical analysis

Statistical analyses were performed using the SPSS 21.0 software (IBM Corp. Armonk, NY, USA). Measurement data are expressed as mean ± standard deviation. The differences between two groups were identified using the independent *t*-test. Data from multiple groups were compared by one-way analysis of variance with Tukey’s post hoc test. A value of *p* < 0.05 indicated statistical significance.

## Supplementary Material

Supplementary Table 1

## References

[1] Finn AV, Nakano M, Narula J, Kolodgie FD, Virmani R. Concept of vulnerable/unstable plaque. Arterioscler Thromb Vasc Biol. 2010; 30:1282–92. 10.1161/ATVBAHA.108.179739 20554950

[2] Moore KJ, Tabas I. Macrophages in the pathogenesis of atherosclerosis. Cell. 2011; 145:341–55. 10.1016/j.cell.2011.04.005 21529710 PMC3111065

[3] Moore KJ, Sheedy FJ, Fisher EA. Macrophages in atherosclerosis: a dynamic balance. Nat Rev Immunol. 2013; 13:709–21. 10.1038/nri3520 23995626 PMC4357520

[4] Zernecke A. Dendritic cells in atherosclerosis: evidence in mice and humans. Arterioscler Thromb Vasc Biol. 2015; 35:763–70. 10.1161/ATVBAHA.114.303566 25675999

[5] Pitt JM, Charrier M, Viaud S, André F, Besse B, Chaput N, Zitvogel L. Dendritic cell-derived exosomes as immunotherapies in the fight against cancer. J Immunol. 2014; 193:1006–11. 10.4049/jimmunol.1400703 25049431

[6] Montecalvo A, Larregina AT, Shufesky WJ, Stolz DB, Sullivan ML, Karlsson JM, Baty CJ, Gibson GA, Erdos G, Wang Z, Milosevic J, Tkacheva OA, Divito SJ, et al. Mechanism of transfer of functional microRNAs between mouse dendritic cells via exosomes. Blood. 2012; 119:756–66. 10.1182/blood-2011-02-338004 22031862 PMC3265200

[7] Bartel DP. MicroRNAs: target recognition and regulatory functions. Cell. 2009; 136:215–33. 10.1016/j.cell.2009.01.002 19167326 PMC3794896

[8] Madrigal-Matute J, Rotllan N, Aranda JF, Fernández-Hernando C. MicroRNAs and atherosclerosis. Curr Atheroscler Rep. 2013; 15:322. 10.1007/s11883-013-0322-z 23512606 PMC4193541

[9] Wei Y, Nazari-Jahantigh M, Neth P, Weber C, Schober A. MicroRNA-126, -145, and -155: a therapeutic triad in atherosclerosis? Arterioscler Thromb Vasc Biol. 2013; 33:449–54. 10.1161/ATVBAHA.112.300279 23324496

[10] Jovanović I, Zivković M, Jovanović J, Djurić T, Stanković A. The co-inertia approach in identification of specific microRNA in early and advanced atherosclerosis plaque. Med Hypotheses. 2014; 83:11–15. 10.1016/j.mehy.2014.04.019 24815336

[11] Nie W, Zhang X, Yan H, Li S, Zhu W, Fan F, Zhu J. Xiaoxianggou attenuates atherosclerotic plaque formation in endogenous high ang II ApoE-^/-^ mice via the inhibition of miR-203 on the expression of ets-2 in endothelial cells. Biomed Pharmacother. 2016; 82:173–79. 10.1016/j.biopha.2016.04.065 27470353

[12] Pan L, Li Y, Jia L, Qin Y, Qi G, Cheng J, Qi Y, Li H, Du J. Cathepsin S deficiency results in abnormal accumulation of autophagosomes in macrophages and enhances ang II-induced cardiac inflammation. PLoS One. 2012; 7:e35315. 10.1371/journal.pone.0035315 22558139 PMC3340377

[13] Samokhin AO, Lythgo PA, Gauthier JY, Percival MD, Brömme D. Pharmacological inhibition of cathepsin S decreases atherosclerotic lesions in Apoe-/- mice. J Cardiovasc Pharmacol. 2010; 56:98–105. 10.1097/FJC.0b013e3181e23e10 20410833

[14] Hsieh MJ, Lin CW, Chen MK, Chien SY, Lo YS, Chuang YC, Hsi YT, Lin CC, Chen JC, Yang SF. Inhibition of cathepsin S confers sensitivity to methyl protodioscin in oral cancer cells via activation of p38 MAPK/JNK signaling pathways. Sci Rep. 2017; 7:45039. 10.1038/srep45039 28327651 PMC5361207

[15] Fan Y, Wang J, Wei L, He B, Wang C, Wang B. Iron deficiency activates pro-inflammatory signaling in macrophages and foam cells via the p38 MAPK-NF-κB pathway. Int J Cardiol. 2011; 152:49–55. 10.1016/j.ijcard.2010.07.005 20674992

[16] Hu Y, Sun B, Liu K, Yan M, Zhang Y, Miao C, Ren L. Icariin attenuates high-cholesterol diet induced atherosclerosis in rats by inhibition of inflammatory response and p38 MAPK signaling pathway. Inflammation. 2016; 39:228–36. 10.1007/s10753-015-0242-x 26307750

[17] Liu L, Zeng P, Yang X, Duan Y, Zhang W, Ma C, Zhang X, Yang S, Li X, Yang J, Liang Y, Han H, Zhu Y, et al. Inhibition of vascular calcification. Arterioscler Thromb Vasc Biol. 2018; 38:2382–95. 10.1161/ATVBAHA.118.311546 30354214

[18] Hotamisligil GS. Endoplasmic reticulum stress and atherosclerosis. Nat Med. 2010; 16:396–99. 10.1038/nm0410-396 20376052 PMC2897068

[19] Kim J, Zhang L, Peppel K, Wu JH, Zidar DA, Brian L, DeWire SM, Exum ST, Lefkowitz RJ, Freedman NJ. Beta-arrestins regulate atherosclerosis and neointimal hyperplasia by controlling smooth muscle cell proliferation and migration. Circ Res. 2008; 103:70–79. 10.1161/CIRCRESAHA.108.172338 18519945 PMC2760159

[20] Nazari-Jahantigh M, Wei Y, Noels H, Akhtar S, Zhou Z, Koenen RR, Heyll K, Gremse F, Kiessling F, Grommes J, Weber C, Schober A. MicroRNA-155 promotes atherosclerosis by repressing Bcl6 in macrophages. J Clin Invest. 2012; 122:4190–202. 10.1172/JCI61716 23041630 PMC3484435

[21] Wang J, Wang WN, Xu SB, Wu H, Dai B, Jian DD, Yang M, Wu YT, Feng Q, Zhu JH, Zhang L, Zhang L. MicroRNA-214-3p: a link between autophagy and endothelial cell dysfunction in atherosclerosis. Acta Physiol (Oxf). 2018; 222:e12973. 10.1111/apha.12973 28888077

[22] Schober A, Nazari-Jahantigh M, Wei Y, Bidzhekov K, Gremse F, Grommes J, Megens RT, Heyll K, Noels H, Hristov M, Wang S, Kiessling F, Olson EN, Weber C. MicroRNA-126-5p promotes endothelial proliferation and limits atherosclerosis by suppressing Dlk1. Nat Med. 2014; 20:368–76. 10.1038/nm.3487 24584117 PMC4398028

[23] Zhou M, Chen J, Zhou L, Chen W, Ding G, Cao L. Pancreatic cancer derived exosomes regulate the expression of TLR4 in dendritic cells via miR-203. Cell Immunol. 2014; 292:65–69. 10.1016/j.cellimm.2014.09.004 25290620

[24] Qin Y, Cao X, Guo J, Zhang Y, Pan L, Zhang H, Li H, Tang C, Du J, Shi GP. Deficiency of cathepsin S attenuates angiotensin II-induced abdominal aortic aneurysm formation in apolipoprotein e-deficient mice. Cardiovasc Res. 2012; 96:401–10. 10.1093/cvr/cvs263 22871592 PMC3500043

[25] Lafarge JC, Naour N, Clément K, Guerre-Millo M. Cathepsins and cystatin C in atherosclerosis and obesity. Biochimie. 2010; 92:1580–86. 10.1016/j.biochi.2010.04.011 20417681

[26] Tang Y, Zheng L, Zhou J, Chen Y, Yang L, Deng F, Hu Y. miR-203-3p participates in the suppression of diabetes-associated osteogenesis in the jaw bone through targeting Smad1. Int J Mol Med. 2018; 41:1595–607. 10.3892/ijmm.2018.3373 29328402 PMC5819914

[27] Adorni MP, Favari E, Ronda N, Granata A, Bellosta S, Arnaboldi L, Corsini A, Gatti R, Bernini F. Free cholesterol alters macrophage morphology and mobility by an ABCA1 dependent mechanism. Atherosclerosis. 2011; 215:70–76. 10.1016/j.atherosclerosis.2010.12.004 21215399

[28] Qin Y, Shi GP. Cysteinyl cathepsins and mast cell proteases in the pathogenesis and therapeutics of cardiovascular diseases. Pharmacol Ther. 2011; 131:338–50. 10.1016/j.pharmthera.2011.04.010 21605595 PMC3134138

[29] de Nooijer R, Bot I, von der Thüsen JH, Leeuwenburgh MA, Overkleeft HS, Kraaijeveld AO, Dorland R, van Santbrink PJ, van Heiningen SH, Westra MM, Kovanen PT, Jukema JW, van der Wall EE, et al. Leukocyte cathepsin S is a potent regulator of both cell and matrix turnover in advanced atherosclerosis. Arterioscler Thromb Vasc Biol. 2009; 29:188–94. 10.1161/ATVBAHA.108.181578 19095996

[30] Cybulsky MI, Cheong C, Robbins CS. Macrophages and dendritic cells: partners in atherogenesis. Circ Res. 2016; 118:637–52. 10.1161/CIRCRESAHA.115.306542 26892963

[31] Gautier EL, Huby T, Saint-Charles F, Ouzilleau B, Pirault J, Deswaerte V, Ginhoux F, Miller ER, Witztum JL, Chapman MJ, Lesnik P. Conventional dendritic cells at the crossroads between immunity and cholesterol homeostasis in atherosclerosis. Circulation. 2009; 119:2367–75. 10.1161/CIRCULATIONAHA.108.807537 19380622

[32] Zhang B, Yin Y, Lai RC, Lim SK. Immunotherapeutic potential of extracellular vesicles. Front Immunol. 2014; 5:518. 10.3389/fimmu.2014.00518 25374570 PMC4205852

[33] Figueiredo JL, Aikawa M, Zheng C, Aaron J, Lax L, Libby P, de Lima Filho JL, Gruener S, Fingerle J, Haap W, Hartmann G, Aikawa E. Selective cathepsin S inhibition attenuates atherosclerosis in apolipoprotein e-deficient mice with chronic renal disease. Am J Pathol. 2015; 185:1156–66. 10.1016/j.ajpath.2014.11.026 25680278 PMC4380840

[34] Zhang A, Lakshmanan J, Motameni A, Harbrecht BG. MicroRNA-203 suppresses proliferation in liver cancer associated with PIK3CA, p38 MAPK, c-Jun, and GSK3 signaling. Mol Cell Biochem. 2018; 441:89–98. 10.1007/s11010-017-3176-9 28887744

[35] Mei S, Gu H, Ward A, Yang X, Guo H, He K, Liu Z, Cao W. P38 mitogen-activated protein kinase (MAPK) promotes cholesterol ester accumulation in macrophages through inhibition of macroautophagy. J Biol Chem. 2012; 287:11761–68. 10.1074/jbc.M111.333575 22354961 PMC3320924

[36] Valadi H, Ekström K, Bossios A, Sjöstrand M, Lee JJ, Lötvall JO. Exosome-mediated transfer of mRNAs and microRNAs is a novel mechanism of genetic exchange between cells. Nat Cell Biol. 2007; 9:654–59. 10.1038/ncb1596 17486113

[37] Li J, Liu K, Liu Y, Xu Y, Zhang F, Yang H, Liu J, Pan T, Chen J, Wu M, Zhou X, Yuan Z. Exosomes mediate the cell-to-cell transmission of IFN-α-induced antiviral activity. Nat Immunol. 2013; 14:793–803. 10.1038/ni.2647 23832071

[38] Xiao J, Pan Y, Li XH, Yang XY, Feng YL, Tan HH, Jiang L, Feng J, Yu XY. Cardiac progenitor cell-derived exosomes prevent cardiomyocytes apoptosis through exosomal miR-21 by targeting PDCD4. Cell Death Dis. 2016; 7:e2277. 10.1038/cddis.2016.181 27336721 PMC5143405

[39] Gong M, Zhuo X, Ma A. STAT6 upregulation promotes M2 macrophage polarization to suppress atherosclerosis. Med Sci Monit Basic Res. 2017; 23:240–49. 10.12659/msmbr.904014 28615615 PMC5484610

[40] Gao W, Liu H, Yuan J, Wu C, Huang D, Ma Y, Zhu J, Ma L, Guo J, Shi H, Zou Y, Ge J. Exosomes derived from mature dendritic cells increase endothelial inflammation and atherosclerosis via membrane TNF-α mediated NF-κB pathway. J Cell Mol Med. 2016; 20:2318–27. 10.1111/jcmm.12923 27515767 PMC5134386

[41] Ayuk SM, Abrahamse H, Houreld NN. The role of photobiomodulation on gene expression of cell adhesion molecules in diabetic wounded fibroblasts *in vitro*. J Photochem Photobiol B. 2016; 161:368–74. 10.1016/j.jphotobiol.2016.05.027 27295416

