# Supplementary material for: Transfer of exosomal microRNA-203-3p from dendritic cells to bone marrow-derived macrophages reduces development of atherosclerosis by downregulating Ctss in mice
Source: Aging (Albany NY). 2021 Jun 2;13(11):15638–58. doi: 10.18632/aging.103842 (PMC8221304; doi:10.18632/aging.103842)
Supplement: Supplementary Table 1 [file aging-13-103842-s001.pdf]

Supplementary Table 1. Information for the DEGs identified from the GSE56143 datasets (|log2FC| > 2, *p* value < 0.05).

Name	logFC	*P* value	Category
Laptm5	3.43302635	0.002335927	Up-regulated gene
Tyrobp	3.072138321	0.001507479	Up-regulated gene
Aif1	2.964939667	0.001504818	Up-regulated gene
Lyz	2.858257818	0.010940116	Up-regulated gene
Mgl1	2.791690124	0.007495697	Up-regulated gene
Cd74	2.695351673	2.00602E-05	Up-regulated gene
H2-Ab1	2.590206533	0.000163343	Up-regulated gene
Lyzs	2.533034755	0.015025385	Up-regulated gene
C1qb	2.490464054	0.002921442	Up-regulated gene
LOC641240	2.413707091	0.000182632	Up-regulated gene
Ctss	2.388743612	0.00187931	Up-regulated gene
H2-Aa	2.343612474	0.000115273	Up-regulated gene
Hist1h3e	2.337675545	0.029259965	Up-regulated gene
Gja1	2.295348155	0.027565406	Up-regulated gene
Lat2	2.215551421	0.00019548	Up-regulated gene
LOC100044702	2.141945425	0.037312241	Up-regulated gene
S100a9	2.131524807	0.013831571	Up-regulated gene
Fcer1g	2.083543016	0.006153202	Up-regulated gene
Mmp12	2.069323539	0.000232579	Up-regulated gene
Rhpn2	-2.037428795	0.005590133	Down-regulated gene
Klk8	-2.087072084	0.016888564	Down-regulated gene
1500015O10Rik	-2.210334088	0.033440755	Down-regulated gene
4Cytl1	-2.310116833	0.038273943	Down-regulated gene
Aldh3a1	-2.374563124	0.0006919	Down-regulated gene
Klk10	-2.480353286	0.000244521	Down-regulated gene
D330027H18Rik	-2.569774264	2.99898E-07	Down-regulated gene
